# A Novel Deep Transfer Learning Method for Intelligent Fault Diagnosis Based on Variational Mode Decomposition and Efficient Channel Attention

**DOI:** 10.3390/e24081087

**Published:** 2022-08-06

**Authors:** Caiming Liu, Xiaorong Zheng, Zhengyi Bao, Zhiwei He, Mingyu Gao, Wenlong Song

**Affiliations:** 1 School of Electronic Information, Hangzhou Dianzi University, Hangzhou 310018, China; 2 Zhejiang Provincial Key Lab of Equipment Electronics, Hangzhou 310018, China; 3 Tianneng Battery Group Co., Ltd., Changxing 313100, China

**Keywords:** deep transfer learning, intelligent fault diagnosis, variational mode decomposition, efficient channel attention

## Abstract

In recent years, deep learning has been applied to intelligent fault diagnosis and has achieved great success. However, the fault diagnosis method of deep learning assumes that the training dataset and the test dataset are obtained under the same operating conditions. This condition can hardly be met in real application scenarios. Additionally, signal preprocessing technology also has an important influence on intelligent fault diagnosis. How to effectively relate signal preprocessing to a transfer diagnostic model is a challenge. To solve the above problems, we propose a novel deep transfer learning method for intelligent fault diagnosis based on Variational Mode Decomposition (VMD) and Efficient Channel Attention (ECA). In the proposed method, the VMD adaptively matches the optimal center frequency and finite bandwidth of each mode to achieve effective separation of signals. To fuse the mode features more effectively after VMD decomposition, ECA is used to learn channel attention. The experimental results show that the proposed signal preprocessing and feature fusion module can increase the accuracy and generality of the transfer diagnostic model. Moreover, we comprehensively analyze and compare our method with state-of-the-art methods at different noise levels, and the results show that our proposed method has better robustness and generalization performance.

## 1. Introduction

Mechanical equipment is one of the most critical components for the normal operation and production of intelligent factories. Due to the long-term use of the equipment and some human factors, the equipment may malfunction, causing major economic losses and personnel injuries. Therefore, fault location and maintenance of mechanical equipment are particularly significant. In practical industrial applications, most equipment fault location and maintenance rely on previous experience or expert knowledge, which requires a lot of manpower and time costs. To solve this problem, intelligent fault diagnosis combines artificial intelligence technology with fault diagnosis technology, which has become an important branch of intelligent health management. Traditional machine learning methods have been widely used in the field of equipment fault diagnosis, such as support vector machine (SVM) [1], random forest (RF) [2], k-nearest neighbor (KNN) [3], and artificial neural network (ANN) [4]. However, these machine learning methods for fault diagnosis rely heavily on industry experts to extract artificial features from raw signals [5]. Moreover, due to the explosive growth of available manufacturing data and the increasing complexity of equipment, traditional machine learning can no longer meet the current requirements for intelligent fault diagnosis.

The application of deep learning has developed rapidly in recent years [6,7]. To solve the above problems, deep learning has been widely used in the field of intelligent fault diagnosis and has achieved fruitful results [8]. Janssens et al. [9] used a convolutional neural network (CNN) to automatically extract the fault features of the original signals, avoiding the trouble of manual feature extraction. Zhao et al. [10] embedded a soft threshold denoising module into a deep residual network. The deep learning diagnostic model has improved feature learning capability for strong noise vibration signals. Nevertheless, the improvement of the soft thresholding function embedded in the network needs further discussion. Lei et al. [11] constructed the long-term dependence of time series captured by long- and short-term memory networks. It is applied to wind turbine condition monitoring and fault diagnosis and has achieved great results. Although the deep learning diagnosis method has a strong feature extraction ability, the model diagnosis performance will be greatly reduced when the training data and test data do not meet the independent and identical distribution conditions. This problem is known as the domain shift problem.

Transfer learning [12,13] improves the diagnostic performance of the diagnostic model in the target domain by learning relevant knowledge from similar scenarios. It provides a new way to solve the domain shift problem. When the data distribution of the source domain and the target domain is different, but the learning task is the same, the transfer learning in this scenario is called domain adaptation. Deep domain adaptation [14,15] combines the powerful feature extraction capability of deep learning. It can effectively learn the domain-invariant representation features of data and enhance the performance of diagnostic models in the target domain. Lei et al. [16] proposed a deep convolutional transfer learning network to explore intelligent fault diagnoses for machines with unlabeled data. The maximum mean difference (MMD) [17] domain discriminator was used to optimize the model and improve accuracy. Jiao et al. [18] proposed a residual joint domain adaptive adversarial network model. Joint maximum mean difference (JMMD) and adversarial domain adaptive discriminator are introduced to align the source and target domain feature spaces. However, the performance of these methods in noisy scenarios needs to be verified. Based on this problem, signal preprocessing in the feedforward network is one of the options for denoising. Qian et al. [19] proposed an unsupervised transfer learning model for a convolutional autoencoder. The Autoencoder is used to denoise the signal. However, the output of the autoencoder is lossy. The removal of noise will also remove some useful weak signals. Li et al. [20] thought from the perspective of a frequency domain. A frequency-domain fusion convolutional neural network for domain adaptive fault diagnosis is proposed. It provides an example adaptation network design for the unified domain but extracting frequency characteristics directly from the CNN still cannot be fully interpreted. Ben Ali et al. [21] proposed an automatic bearing fault diagnosis method based on empirical mode decomposition (EMD) to successfully detect the severity of defects online. However, determining the intrinsic mode functions (IMFs) best suited for bearing fault diagnosis requires expert experience. DASENet achieves high accuracy with few input data points [22]. The inputs to this model are the frequency domain signal and the time-frequency graph signal. In addition, it fuses features through the squeeze-and-excitation attention (SEA) module, which improves the generality of the diagnostic model. Nevertheless, this method incorporates the features of duplication and is not a transfer diagnostic model. Wu et al. [23] introduced ensemble empirical mode decomposition (EEMD) signal preprocessing and attention mechanisms into the transfer diagnosis model to extract domain-invariant features. In their study, the signal preprocessing part is just a function of denoising. In addition, the diagnosis results are effective, indicating that signal preprocessing is necessary. Although the above methods integrate signal preprocessing technology into deep learning or transfer learning methods to improve the performance of diagnostic models, there are still several open questions, and the following situations should be considered.

1. Due to differences in the distribution of acquired vibration data, the effectiveness of the deep learning model diagnosis method is limited. Therefore, it is necessary to introduce signal preprocessing into the transfer learning diagnostic model.
2. The original vibration signal is a typical non-stationary signal [24]. However, the commonly used Fourier transform method has insufficient processing ability for non-stationary signals (signals whose frequencies change with time). Although the EMD method eliminates the limitation of Fourier transform, it also has a serious mode aliasing problem.
3. The signal preprocessing method and the intelligent diagnosis model based on transfer learning are not effectively related. An effective connection module is required between them instead of being directly preprocessed into new data for input.

To bridge these gaps, this paper proposes a novel deep transfer learning method for intelligent fault diagnosis based on Variational Mode Decomposition and Efficient Channel Attention. The corresponding framework of this method is divided into three parts: signal preprocessing using VMD, mode feature fusion using ECA, and a deep transfer network for intelligent fault diagnosis. Since different modes represent signals with different center frequencies, it makes sense to re-weight the channel features. Moreover, joint domain adaptation is used to align the joint distribution of input features and output labels in the source domain and the target domain to achieve an efficient intelligent fault diagnosis. We comprehensively analyze and compare our method with state-of-the-art methods, and the results show that our proposed method has better robustness and generalization performance. The novelty and main contributions of this paper can be summarized as follows:

1. The signal preprocessing of the VMD is applied for the transfer fault diagnosis. The VMD algorithm is used to decompose original fault vibration signals into a series of intrinsic mode functions (IMFs) with specific bandwidth so that the transfer learning model can learn fault features better.
2. To fuse the mode features after VMD decomposition more effectively, ECA is used to learn channel attention. This module avoids dimensionality reduction and effectively captures cross-channel interactions. Since different modes represent signals with different center frequencies, it makes sense to re-weight the channel features.
3. A novel deep transfer learning method is proposed for intelligent fault diagnosis based on Variational Mode Decomposition and Efficient Channel Attention (VMD-ECA-DTN). Considering that it can accurately fit the domain-invariant representation of fault features, our proposed method has better performance (robustness and generalization) in transfer diagnosis tasks compared with the state-of-the-art methods.

The remainder of this paper begins with the related preliminaries in Section 2. Section 3 introduces the construction of the VMD-ECA-DTN and its optimization objective. In Section 4, the experiments and analyses of the study are given in Section 4. Finally, the conclusions are drawn in Section 5.

## 2. Preliminaries

### 2.1. Domain Adaptation Problem

**Figure 1:**
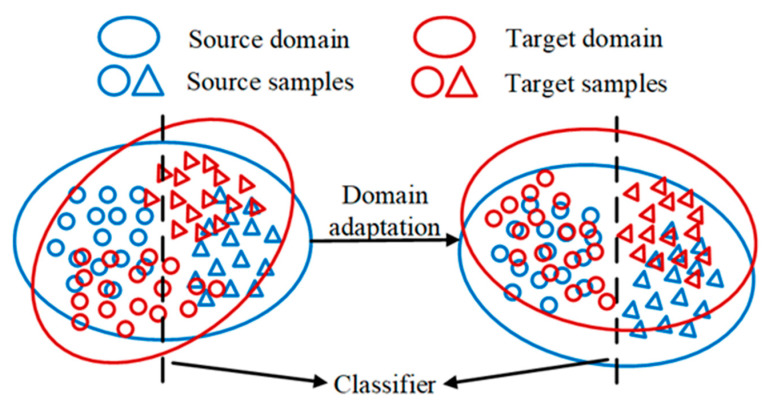
Domain adaptation. The target classifier on the source domain can also be applied to the target domain after the alignment of the domain adaptation feature space.

Domain adaptation refers to transfer learning in a scenario where the source domain and the target domain have different data distribution and the same learning task. The source domain is defined as 𝒟*_s_* = {($x_{1}^{s}$,$y_{1}^{s}$), …, ($x_{ns}^{s}$,$y_{ns}^{s}$)}, and the target domain is defined as 𝒟*_t_* = {($x_{1}^{t}$,$y_{1}^{t}$), …, ($x_{nt}^{t}$,$y_{nt}^{t}$)}, Where *n_s_* is the sample number in the source domain, *n_t_* is the sample number in the target domain. The target domain under unsupervised domain adaptation has no label, and the target domain is de fined as 𝒟*_t_* = {($x_{1}^{t}$), …, ($x_{nt}^{t}$)}. In this paper, the problem of unsupervised domain adaptation fault diagnosis is mainly studied. As shown in Figure 1, the main goal of domain adaptation is to align the feature space of the source. Domain and the target domain to realize cross-domain intelligent fault diagnosis.

### 2.2. Variational Mode Decomposition

The fault vibration signal of equipment is a typical non-stationary signal. To better learn the fault features of the original signal, we use the VMD method to decompose fault vibration signals during signal preprocessing. The VMD algorithm is a completely non-recursive signal decomposition method that decomposes time series to obtain relatively stable sub-signals containing multiple different frequency scales [25,26,27]. This algorithm assumes that all components are narrowband signals concentrated near their respective central frequencies. Therefore, VMD establishes constrained optimization problems according to component narrowband conditions. Then, the center frequency of the signal component is estimated, and the corresponding component is reconstructed. The constrained variational problem is expressed as follows:

(1) $$\begin{array}{r} \min_{\left(u_{k},w_{k}\right)}\left\{{\sum_{k=1}^{K}\|\partial t[(\delta (t)+\frac{j}{\pi t})*u_{k}(t)]e^{-jw_{k}t}\|}_{2}^{2}\right\}\\ s.t.x(t)=\sum_{k=1}^{K}u_{k} \end{array}$$

where *K* indicates the number of modes. {*u_k_*}: = {*u*_1_, …, *u_K_*} represents the set of all modes. {*w_k_*}: = {*w*_1_, …, *w_K_*} represents the set of their center frequencies. *δ*(*t*) is the Dirac distribution, and (*) defines the convolution operation. *x*(*t*) represents the time series of the fault vibration signal. To solve the above mathematical problem, the equation can be equivalent to an unconstrained optimization problem by means of augmented Lagrange functions, as shown in:

(2) $$\begin{array}{r} L(u_{k},w_{k},\lambda )=\alpha {\sum_{k=1}^{K}\|\partial t[(\delta (t)+\frac{j}{\pi t})*u_{k}(t)]e^{-jw_{k}t}\|}_{2}^{2}\\ +{\|x(t)-\sum_{k=1}^{K}u_{k}(t)\|}_{2}^{2}+\langle \lambda (t),x(t)-\sum_{k=1}^{K}u_{k}(t)\rangle \end{array}$$

where *α* is the quadratic penalty parameter, and *λ* is Lagrangian multipliers.

The minimization scheme of the original problem is solved by the alternate direction method of multipliers (ADMM), which keeps the other two variables constant and updates one of them. Therefore, the update formulas for *u_k_* and *w_k_* are expressed as follows:

(3) $${\hat{u}}_{k}^{n+1}=\frac{\hat{x}(w)-\sum_{i>k}{\hat{u}}_{i}(w)-\sum_{i<k}{\hat{u}}_{i}(w)+\frac{\hat{\lambda }(w)}{2}}{1+2\alpha {\left(w-w_{k}\right)}^{2}}$$

(4) $$w_{k}^{n+1}=\frac{\int_{0}^{\infty }w|{{\hat{u}}_{k}^{n+1}(w)|}^{2}dw}{\int_{0}^{\infty }|{{\hat{u}}_{k}^{n+1}(w)|}^{2}dw}$$

where *n* is the number of iterations. (^) denotes the Fourier transform. *w* ≥ 0. The result of solving *u_k_* is a Wiener filter, and the result of solving *w_k_* is a barycentric method of solving signal bandwidth. Given precision *ε*, iteration stops when the following convergence criterion is reached:

(5) $$\sum_{k=1}^{K}{\left\|{\hat{u}}_{k}^{n+1}-{\hat{u}}_{k}^{n}\right\|}_{2}^{2}/{\left\|{\hat{u}}_{k}^{n}\right\|}_{2}^{2}<\varepsilon$$

Finally, all modes of signal decomposition are obtained through the above iterative updating formula. The VMD algorithm is used to decompose original fault vibration signals into a series of intrinsic mode functions (IMFs) with specific bandwidth so that the transfer learning model can learn fault features better.

### 2.3. Efficient Channel Attention

**Figure 2:**
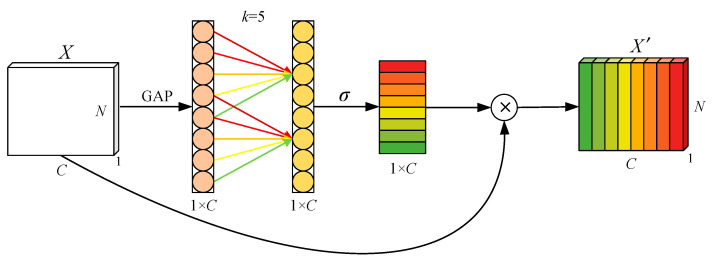
Diagram of efficient channel attention.

The channel attention mechanism has proven to have great potential for improving the performance of deep convolutional neural networks (CNN). Efficient Channel Attention (ECA) [28] is improved based on Squeeze-and-Excitation Attention (SEA) [29]. First, the ECA module aggregates convolution features using global average pooling (GAP) without dimensionality reduction. Second, kernel size *k* is determined adaptively. Then, a one-dimensional convolution operation is performed, and finally Sigmoid function was performed to learn channel attention. The diagram of the ECA is shown in Figure 2:

Let the input be *X*, and the output *X*′ of the ECA is expressed as:

(6) $$X^{\prime }=\sigma ({C1D}_{k}(g(X)))X$$

where C1D indicates one-dimensional convolution, and *k* is the kernel size. *g*(*X*) represents the global average pooling function.

The ECA module uses the 1D convolution layer directly after the global average pooling layer, removing the full connection layer. This module avoids dimensionality reduction and effectively captures cross-channel interactions. In addition, ECA involves only a few parameters to achieve good results. Different from general networks, ECA’s main role in the study is to fuse the features of different modes after VMD decomposition.

## 3. The Proposed VMD-ECA-DTN Method

**Figure 3:**
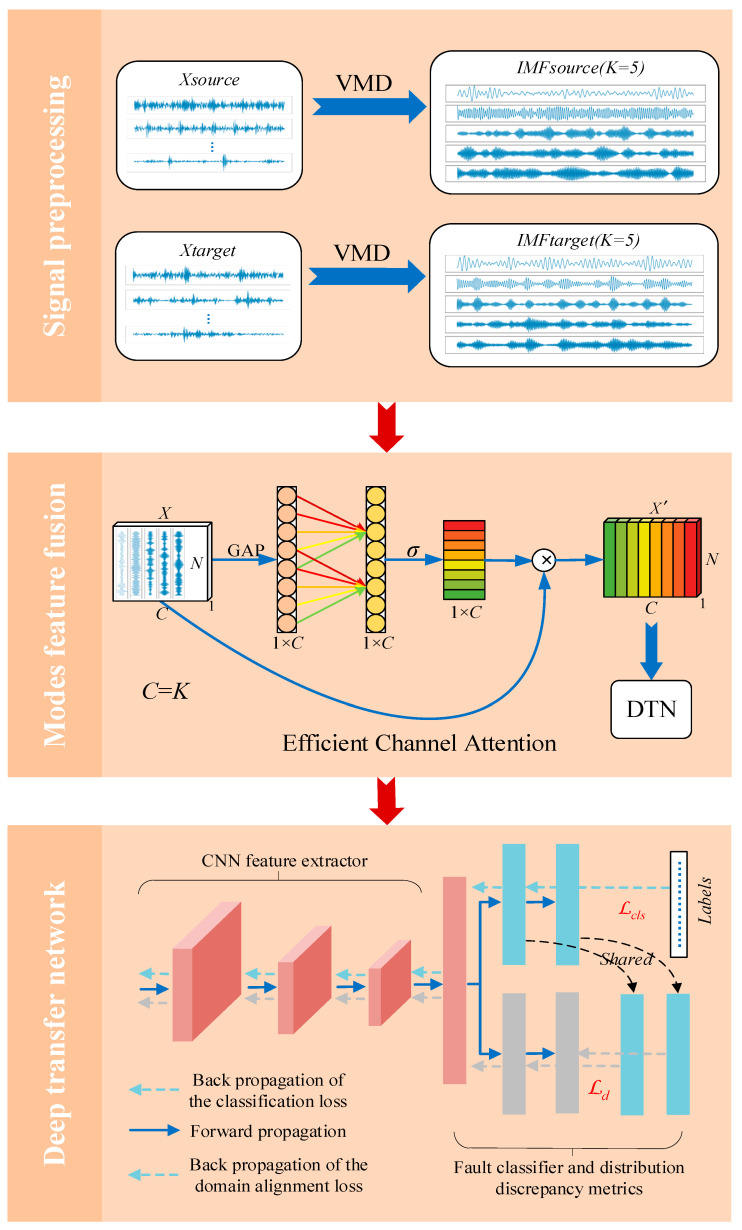
The architecture of VMD-ECA-DTN for intelligent fault diagnosis.

In this section, an advanced and novel deep transfer learning method for intelligent fault diagnosis based on Variational Mode Decomposition and Efficient Channel Attention is proposed. As shown in Figure 3, the corresponding framework of this method is divided into three parts: signal preprocessing using VMD, mode feature fusion using ECA, and a deep transfer network for intelligent fault diagnosis. Signal preprocessing is a significant step in fault analysis. The VMD method adaptively matches the optimal center frequency and finite bandwidth of each mode. The effective separation of IMFs and the frequency domain division of signals are realized. Finally, the effective decomposition components of a given signal are obtained. To fuse the mode features more effectively after VMD decomposition, ECA is used to learn channel attention. Since different modes represent signals with different center frequencies, it makes sense to re-weight the channel features. The last deep transfer network includes a convolution feature extractor, a fault classifier for prediction, and distribution discrepancy metrics with joint domain adaptation to reduce the difference in the joint distribution of input features and output tags between the source domain and the target domain. The goal of the proposed approach is to achieve a better cross-domain device intelligent fault diagnosis by enhancing mode features, which are more favorable for the diagnosis task. It should be noted that the source of training data has two parts: one is the source domain data with labels, and the other is the target domain data without labels. The processing operations of these two parts of data in the signal preprocessing and mode feature fusion stages are similar. As detailed in the following subsections.

### 3.1. Architecture of the Proposed VMD-ECA-DTN

**Figure 4:**
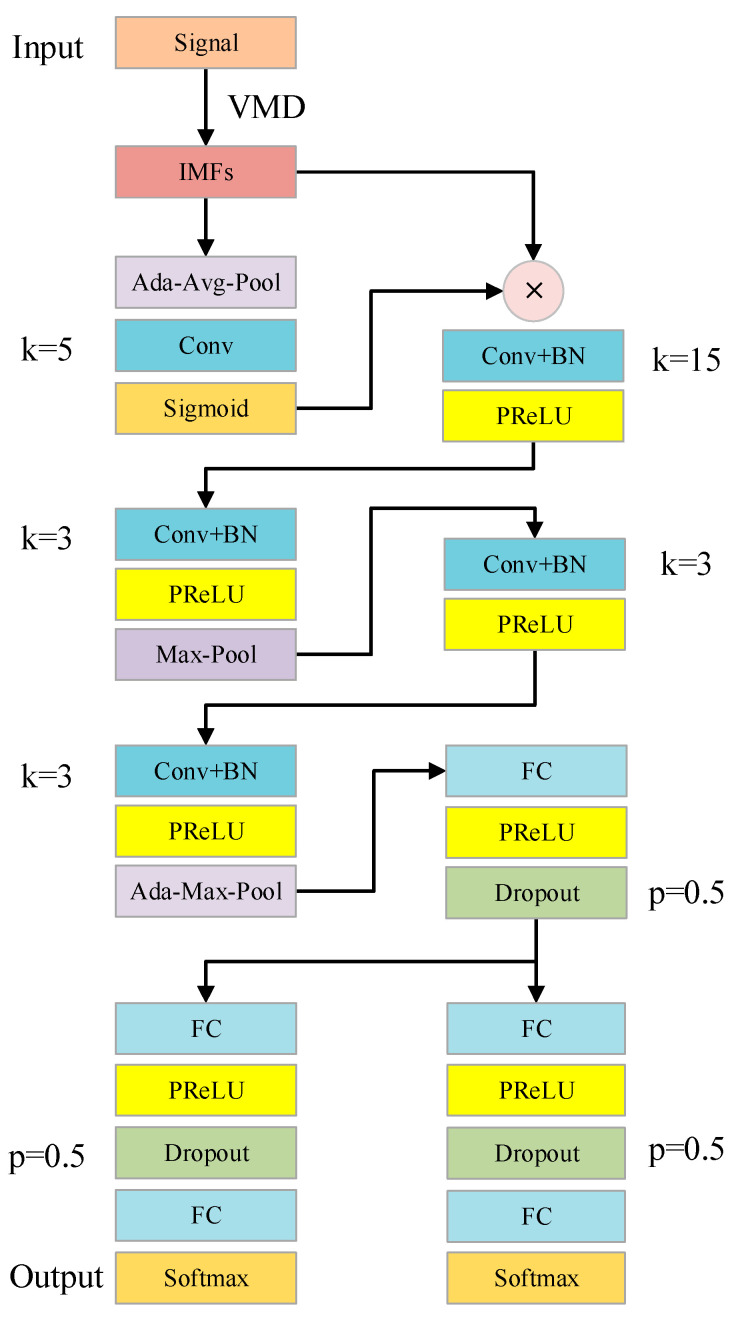
Details of the designed VMD-ECA-DTN architecture.

In this subsection, the specific architecture of the proposed method will be explained in detail. Inspired by a deep transfer network with joint distribution adaptation, the proposed deep transfer network architecture is designed based on VMD and ECA. The detailed architecture of the proposed method is shown in Figure 4. In a general way, the nonlinear correction unit function Parametric Rectified Linear Unit (PRELU) [30] is adopted for the nonlinear activation layer. Given a signal length of 1024 for a single sample, the input shape of the original vibration signal is (64, 1, 1024). 64 indicates the batch size, and 1 indicates the channel size. Set the number of modes of the VMD algorithm as 5, because the length of the mode component obtained after VMD decomposition is consistent with the original signal, the shape of the IMF signal is (64, 5, 1024). To fuse mode features more effectively, the ECA is used to learn channel attention. The ECA block consists of an adaptive global average pooling layer (Ada-Avg-Pool), a convolution layer (Conv), and a sigmoid layer. In addition, there is a weight multiplier at the end. Notably, it does not reduce dimension and effectively captures information about cross-channel interactions. The shape of the signal does not change as it passes through the ECA module, which simply re-weights the mode’s features. Next is the CNN feature extractor, which consists of four convolution layers containing batch normalization (BN). The convolution kernel size of these convolution layers is 15, 3, 3, and 3, respectively. In particular, the reason for using large convolution kernels in the first layer is to increase the receptive field. Inspired by Alexnet, max pooling (Max-Pool) is adopted for the second convolution layer, and adaptive global max pooling (Ada-Max-Pool) is adopted for the fourth convolution layer. To prevent overfitting, we use the dropout trick in the later fully connected layers (FC) as an alternative form of regularization. Set the dropout ratio to p, where each output node is set to zero with probability p. In addition, the architecture of the fault classifier and the distribution discrepancy metrics is similar. The specific parameters of the proposed network architecture are shown in Table 1.

### 3.2. Optimization Objective of VMD-ECA-DTN

Given the mode’s signal set *X* = {*u*_1_, …, *u_K_*} after VMD decomposition, the output *X*′ = *σ*(C1D*_k_*(*g*(*X*)))*X* of ECA is obtained by modal feature fusion based on the attentional mechanism module. The size of the feature map does not change after the ECA module, but the importance of each channel feature has changed. Let *x^l^* be the input of the *l*th convolution layer, and then the feature map after the convolution operation and pooling operation can be expressed as Formula (7):

(7) $$x_{conv}^{l}=pool(\sum K^{l}*x^{l}+b^{l})$$

where *l* is the *l*th convolution layer; *x^l^* is the input; *K^l^* denotes the convolution kernel; *b^l^* denotes the bias. The feature extractor has four convolution layers, and the convolution calculation method is consistent with Formula (7).

According to the structure of the universal classifier, the cross-entropy loss function is used for the fault classifier. We are given the source sample labels. *y_i_* ∈ {1, 2, 3, …, *m*}, *m* denotes the total number of sample categories (including normal categories and fault categories). Therefore, the classification loss can be expressed as follows:

(8) $${ℒ}_{cls}=-\frac{1}{n}(\sum_{i=1}^{n}\sum_{j=1}^{m}I(y_{i}=j)\log(p_{j}))$$

where *N* is the sample batch size of the source domain during training; *p_j_* denotes the probability that the sample is predicted to be *j*th class. *I* is a judgment function that outputs 1 if the input is true and 0 otherwise.

To align the joint distribution of input features and output labels in the source and target domains, the joint maximum mean distance (JMMD) [31] is used for distribution discrepancy metrics. The calculation of this loss function is expressed by:

(9) $${ℒ}_{d}={\left\|E_{x_{s}\sim P(x)}[{⊗}_{l=1}^{m}\phi^{l}(x_{s}^{l})]-E_{x_{t}\sim Q(x)}[{⊗}_{l=1}^{m}\phi^{l}(x_{t}^{l})]\right\|}_{{⊗}_{l=1}^{m}H^{l}}^{2}$$

where *P*(*x*) denotes the feature space distribution of the source domain samples. *Q*(*x*) represents the feature spatial distribution of the target domain samples. ${⊗}_{l=1}^{m}$*ϕ^l^*($x_{s}^{l}$) = *ϕ*^1^($x_{s}^{1}$)⊗*ϕ*^2^($x_{s}^{2}$)⊗···⊗*ϕ^l^*($x_{s}^{l}$) is the feature mapping of the source domain in the tensor product Hilbert space. *l* is the set of higher network layers; ${⊗}_{l=1}^{m}$*ϕ^l^*($x_{t}^{l}$) is the feature mapping of the source domain in the tensor product Hilbert space; ${⊗}_{l=1}^{m}$H*^l^* denotes an *m*th order tensor product feature space.

Therefore, the total loss function can be composed of fault classification loss and domain alignment loss, as shown below:

(10) $$ℒ(\theta_{E},\theta_{F},\theta_{C},\theta_{D})={ℒ}_{cls}+\lambda {ℒ}_{d}$$

where *θ_E_*, *θ_F_*, *θ_C_*, and *θ_D_* represent the parameters of the ECA module, CNN feature extractor, fault classifier, and distribution discrepancy metrics, respectively. *λ* is the weighting parameter. The training process of VMD-ECA-DTN consists of two processes: pre-training and cross-domain adaptation. During the source domain pre-training, fault classification loss is used to optimize the goal. In domain adaptation training, fault classification loss and domain confusion loss are used to optimize the target. Based on Formula (10), the parameters *θ_E_*, *θ_F_*, *θ_C_*, and *θ_D_* are updated in the following order:

(11) $$\theta_{E}\leftarrow \theta_{E}-\mu (\frac{\partial {ℒ}_{cls}}{\partial \theta_{E}}+\lambda \frac{\partial {ℒ}_{d}}{\partial \theta_{E}})$$

(12) $$\theta_{F}\leftarrow \theta_{F}-\mu (\frac{\partial {ℒ}_{cls}}{\partial \theta_{F}}+\lambda \frac{\partial {ℒ}_{d}}{\partial \theta_{F}})$$

(13) $$\theta_{C}\leftarrow \theta_{C}-\mu \frac{\partial {ℒ}_{cls}}{\partial \theta_{C}}$$

(14) $$\theta_{D}\leftarrow \theta_{D}-\mu \frac{\partial {ℒ}_{d}}{\partial \theta_{D}}$$

where *μ* is the learning rate.

## 4. Experimental Results and Analysis

### 4.1. Dataset Introduction and Experiment Setup

To demonstrate the effectiveness of the proposed method, in this section, we select the CWRU bearing fault dataset for experiments, which is a widely used dataset [32]. It was provided by the Case Western Reserve University laboratory and is currently the most used open-source dataset. We selected the driver end fault data with a sampling frequency of 12 kHz and normal sample data. The faults are divided into inner ring faults (IF), outer ring faults (OF), and rolling body faults (RF). The diameter of the fault damage is 7 mils, 14 mils, and 21 mils (1 mil = 0.001 inches). There are 10 categories of fault samples plus normal samples, and 260 samples are selected for each category. According to load and speed, it is divided into four diagnostic tasks, as shown in Table 2. Cross-domain diagnosis tasks 0-1 indicate that the slave source domain is the condition where task code 0 resides, and the target domain is the condition where task code 1 resides. There are 6 cross-domain diagnosis tasks in pairs.

The data is augmented by partial resampling techniques. Each sample of the training set and testing set is a continuous time series with a length of 1024. The dataset for the whole experiment includes data collected under three different working conditions. According to different working conditions, the data are divided into source domain data and target domain data. The source domain data contain 1000 samples, and the target domain data contan 1600 samples. Source domain data have labels, but target domain data have no labels. The details of the number of samples in the training dataset, validation dataset, and testing dataset are given in Table 3. x − y indicates that the source domain is x, and the target domain is y.

During the following comparative experiment, all calculation was carried out on a computer with Intel(R) Core(TM) i7-6700K (4.00 GHz), NVIDIA GTX 1080Ti graphics cards, 64 GB RAM and Pytorch framework. To reduce the impact of accidental behavior in the experiment, all classification processes were repeated 10 times, and the trimmed mean value was taken as the final result (average after removing the highest and lowest scores). The max training epoch is set to 200, with a batch size of 64. In practical applications, the original vibration data collected by sensors are often mixed with noise, so the intelligent fault diagnosis models is required to have a certain anti-noise ability. To demonstrate the anti-noise capability of the proposed method, various Gaussian noises were added to the original vibration signal, and SNR [33] was used to evaluate the noise level, which was defined as:

(15) $$\mathrm{SNR}(dB)=10\cdot \mathrm{lg}(P_{sig\mathrm{na}l}/P_{noise})$$

where *P_signal_* and *P_noise_* represent the effective power of the raw vibration signal and noise signal, respectively.

### 4.2. Comparison of Signal Preprocessing Methods

Signal preprocessing techniques play an important role in intelligent fault diagnosis. The effective signal preprocessing method is beneficial to the intelligent diagnosis model for better learning the fault features in the original signal. To illustrate the effectiveness and advantages of the proposed VMD preprocessing method in the whole intelligent fault diagnosis framework, popular preprocessing methods are compared. Notably, all comparison methods are consistent with the rest of the framework, except for signal preprocessing. All details of the contrastive methods are illustrated as follows:

1. FFT [34]: The basic idea of FFT (Fast Fourier Transform) is to decompose the original sequence of N points into a series of short sequences.
2. EMD [35]: EMD (Empirical Mode Decomposition) is a signal processing method in the time-frequency domain, which is based on the time-scale characteristics of the data itself, without setting any basis function in advance. EMD has obvious advantages in processing non-stationary and nonlinear data and is suitable for analyzing nonlinear non-stationary signal sequences with a high signal-to-noise ratio.
3. VMD: The signal decomposition method we use in the signal preprocessing stage. It shifts the acquisition of signal components into a variational framework. A non-recursive processing strategy is used to decompose the original signal by constructing and solving a constrained variational problem, which can effectively avoid problems such as modal aliasing, over-envelope, under-envelope, and boundary effects.

**Figure 5:**
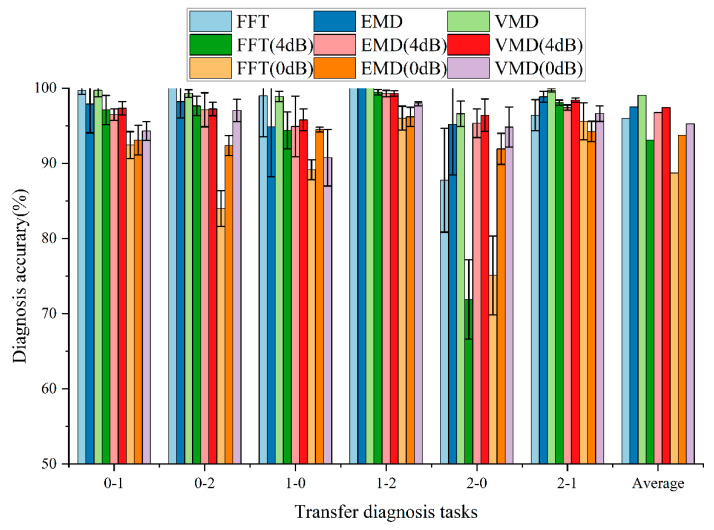
Comparison results of signal preprocessing methods under different transfer diagnosis tasks.

In addition, Gaussian noises of 4 dB and 0 dB were added to the original vibration signal, respectively. The purpose of adding noise is to verify the anti-noise and robustness of the signal preprocessing method. The comparison results of the signal preprocessing methods under different noise levels are illustrated in Figure 5 and Table 4.

From the comparison results, we can draw the following conclusions:

1. When the original vibration signal is not added with Gaussian noise, the average accuracy of all signal preprocessing methods for the six transfer diagnosis tasks is more than 97.00%. After Gaussian noise is added to the original vibration signal, the diagnostic accuracy of all signal preprocessing methods is almost reduced. This shows that the addition of noise seriously affects the performance of the model. In addition, the higher the noise level, the more serious the performance of the intelligent fault diagnosis model decreases.
2. At the same noise level, the FFT preprocessing method has the worst performance, while the VMD preprocessing method has the best performance. Although the VMD preprocessing method is not optimal in individual transfer diagnosis tasks (such as 0-2 transfer diagnosis task without adding noise), the VMD preprocessing method has the highest cross-domain diagnosis accuracy in most transfer diagnosis tasks as a whole. The results show that VMD preprocessing can provide better anti-noise and robustness for intelligent fault diagnosis models.

**Figure 6:**
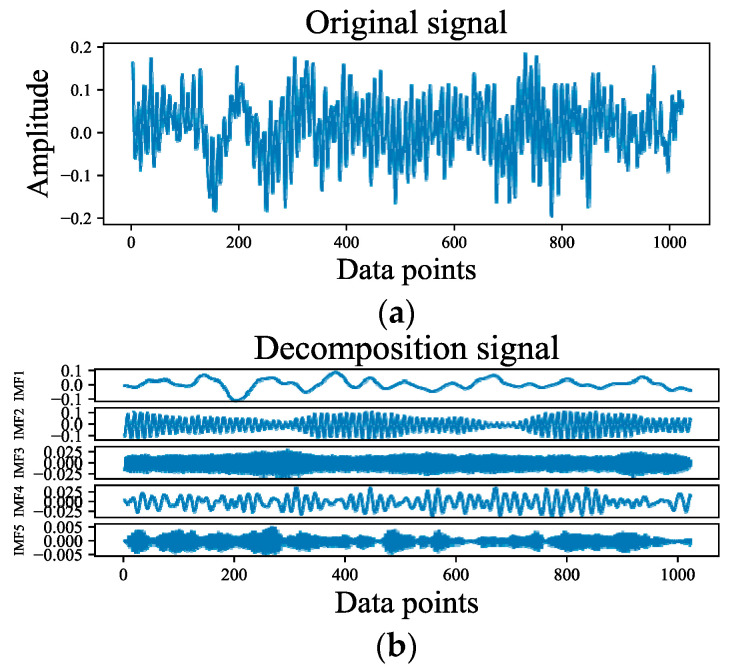

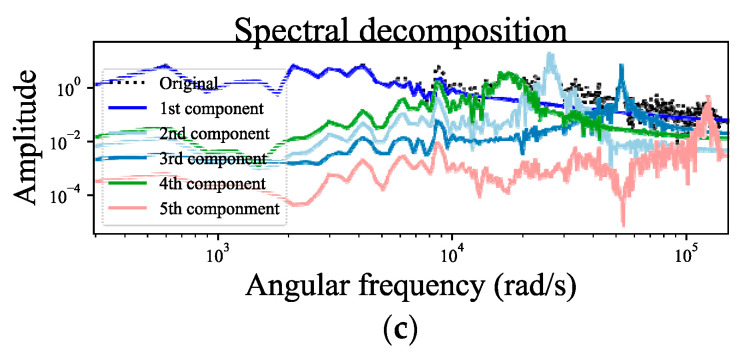
Diagram of signal decomposition. (**a**) Original signal; (**b**) Decomposition signal; (**c**) Spectral decomposition.

**Figure 7:**
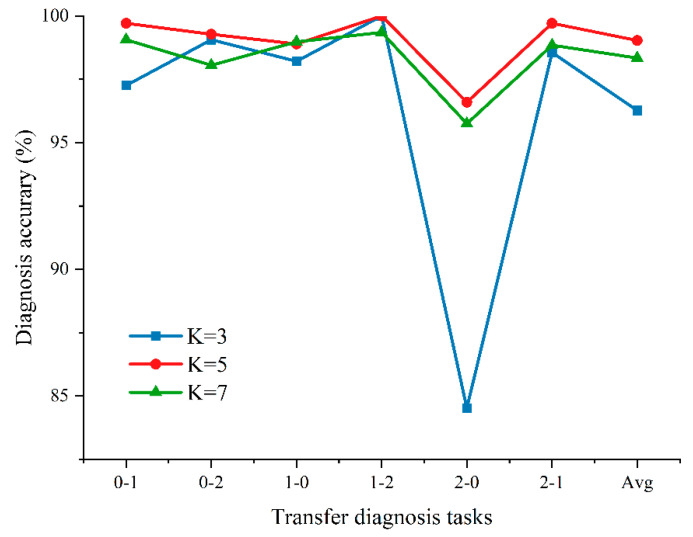
Experimental results with different hyperparameters *K*.

Based on the above two points, it can be concluded that the VMD preprocessing method has a beneficial effect on neural network learning fault features. The original signal, decomposition signal, and signal center frequency are shown in Figure 6. This shows that the different modes of decomposition represent the different central frequency characteristics of the fault signals. Decomposition of the signal will be more conducive to intelligent model learning and will play a role in noise reduction. Notably, the number of modes *K* is a hyperparameter. The effect of VMD decomposition is mainly affected by the selected value of the modal number. When the selected value of the mode is small, since the VMD algorithm is equivalent to an adaptive filter bank, some important information in the original signal will be filtered, affecting the subsequent feature extraction. When the selected value of the mode is large, the center frequencies of the adjacent mode components will be close to each other, resulting in mode repetition or additional noise. The main difference between the different modes is the difference in center frequency. Therefore, the appropriate mode value is selected by observing the distribution of the center frequency under different mode numbers. We set *K* equal to 5 by this method. In addition, we conducted some experiments on different hyperparameters *K*, as shown in Figure 7. This indicates that the VMD algorithm needs an appropriate *K* setting.

### 4.3. Comparison of Feature Fusion Methods

**Figure 8:**
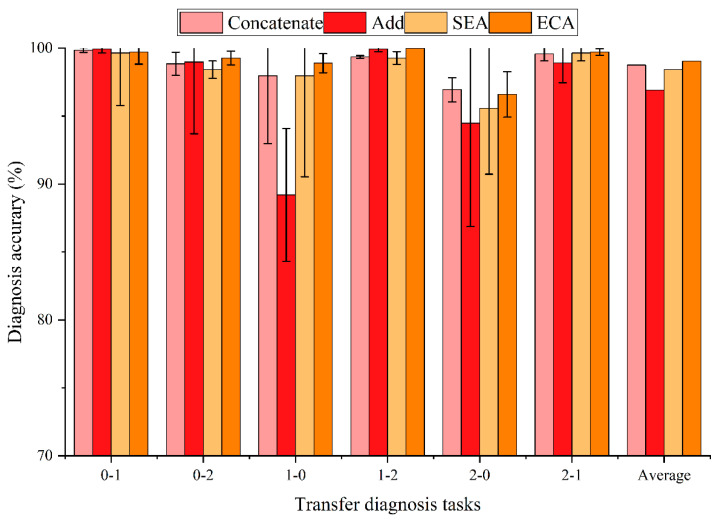
Comparison results of different feature fusion methods.

How to fuse the decomposed mode signal features is a difficult problem. After the original signal decomposition, each mode represents the fault features of different central frequencies. The aim of adding channel attention mechanisms to the intelligent fault diagnosis model is to reweight and fuse different mode features. This means that the transfer learning fault diagnosis network automatically learns which modes are more important to the final objective optimization. To illustrate the effectiveness and advantages of the proposed ECA feature fusion method in the whole intelligent fault diagnosis framework, popular feature fusion methods are compared. Notably, all comparison methods are consistent with the rest of the framework, except for feature fusion. We used Concatenate, Add, SEA, and ECA (the attentional mechanism we use in the proposed transfer learning framework) to conduct experiments for comparison. The experimental results are shown in Figure 8 and Table 5.

The experimental results show that different feature fusion methods have different effects on transfer fault diagnosis. However, the effect of feature fusion is not as important as signal preprocessing. In addition, the feature fusion method needs to be combined with the pretreatment method. In this way, the decomposed modes of VMD can be better utilized. As can be seen from Table 5, the ECA feature fusion method achieves the highest average diagnostic accuracy. In addition, the accuracy standard deviation of the ECA method is relatively lower.

### 4.4. Comparison between the Proposed Method and State-Of-The-Art Methods

To verify the universality and effectiveness of the proposed VMD-ECA-DTN, we compared it with state-of-the-art methods. The methods to be compared are WDCNN (Wide Deep Convolutional Neural Networks) [36], DDC (Deep Domain Confusion Network) [37], DANN (Deep Adversarial Neural Network) [38], and DTN (Deep Transfer Network with joint distribution adaptation) [39]. In particular, the WDCNN model is trained only by the labeled samples in the source domain, while the other models are trained by a combination of labeled samples in the source domain and unlabeled samples in the target domain. To facilitate comparison, the structural design of these models adopts a CNN feature extractor. The number of network layers inside CNN is consistent with the proposed VMD-ECA-DTN, and the hyperparameters of these models are also consistent with the VMD-ECA-DTN. Moreover, to comprehensively analyze and compare all methods objectively, all classification processes were repeated 10 times, and the trimmed mean value was taken as the result. The comparison results of the proposed method with other state-of-the-art methods are shown in Table 6.

From the comparison results above, we can draw the following conclusions:

1. Among the five methods, only WDCNN is not a method of domain adaptation, and its average classification accuracy is lower than that of the other four methods, indicating the effectiveness of the domain adaptive method for cross-domain diagnosis. After adding 0 dB noise to the original data, the average diagnostic accuracy of WDCNN is only 93.31%.
2. Adding Gaussian noise to the original signal affects the ability of the intelligent fault diagnosis model to learn fault features. The proposed method achieves the best average performance in different noise environments. Even under 0 dB noise, the average diagnostic accuracy of the proposed method is 95.24%. This shows that the proposed method has better robustness and universality.
3. In a specific transfer diagnostic task, the performance of the proposed method is not the best. For example, in the 0-1 diagnostic task with 0 dB noise, the diagnostic accuracy of the proposed method is only 90.73%. This may indicate that different levels of noise will affect VMD signal preprocessing, and thus affect neural network learning fault features. However, in most transfer diagnosis tasks, our proposed method is superior to other state-of-the-art methods. This again shows the effectiveness and robustness of our proposed method.

## 5. Conclusions

In this study, we propose a novel deep transfer learning method for intelligent fault diagnosis based on Variational Mode Decomposition and Efficient Channel Attention. VMD signal preprocessing is used to decompose the original vibration signals into mode signals of different center frequencies. The effective separation of IMFs and the frequency domain division of signals are realized. Finally, the effective decomposition components of a given signal are obtained. To fuse the mode features more effectively after VMD decomposition, ECA is used to learn channel attention. It is used to adaptively learn the importance of different mode features for transfer diagnostic tasks, thereby improving model performance. We study the effects of signal preprocessing, feature fusion, and synthesis on the diagnosis results in the task of transfer diagnosis. The conclusions are as follows:

1. An appropriate signal preprocessing method is beneficial to the transfer diagnosis model. The purpose of preprocessing is to denoise the signal and extract the frequency signal, which is useful for diagnostic tasks.
2. Feature fusion is an important step in learning the main mode features. Compared with other fusion methods, the ECA module integrates the features of different modes, which facilitates the further learning of vibration signals by the diagnostic model.
3. The combination of signal preprocessing and attention mechanisms can be used to extract meaningful features. Our proposed method has better performance (robustness and generalization) in transfer diagnosis tasks compared with state-of-the-art methods.

It has certain application potential in cross-domain intelligent fault diagnosis. However, there are also some shortcomings. First, it is difficult to obtain balanced datasets in practical applications. How to improve the method for imbalanced datasets remains to be discussed. Second, the hyperparameters of VMD preprocessing in this method are still determined by expert experience. Getting rid of additional interventions remains difficult.

In the future, it is still a challenge to popularize intelligent fault diagnosis models in battery manufacturing, aerospace, and other industrial fields. For single application scenarios, such as battery manufacturing equipment, an intelligent fault diagnosis model based on unsupervised transfer learning is feasible. However, it is very difficult to solve the problem of fault diagnosis from laboratory data to actual equipment data. The actual data collected in the industry may be unlabeled and almost glitch-free. The data without any labels are useless for an intelligent model. It takes a long way to get there.

## Figures and Tables

**Table 1:** Specific parameters of the proposed network architecture.

Type	Layer	Kernel Size	Stride	Channel Size	Input Size	Output Size
Input	Reshape	/	/	/	/	(64, 1, 1024)
VMD	VMD output	/	/	/	/	(64, 5, 1024)
ECA	Ada-Avg-Pool	/	/	/	(64, 5, 1024)	(64, 5, 1)
	Reshape	/	/	/	(64, 5, 1)	(64, 1, 5)
	Conv	1	1	1	(64, 1, 5)	(64, 1, 5)
	Sigmoid	/	/	/	(64, 1, 5)	(64, 1, 5)
	Reshape	/	/	/	(64, 1, 5)	(64, 5, 1)
	Multiplier	/	/	/	(64, 5, 1) (64, 1, 1024)	(64, 1, 1024)
CNN feature extractor	Conv	15	1	16	(64, 1, 1024)	(64, 16, 1010)
	BN	/	/	/	(64, 16, 1010)	(64, 16, 1010)
	Conv	3	1	32	(64, 16, 1010)	(64, 32, 1008)
	BN	/	/	/	(64, 32, 1008)	(64, 32, 1008)
	Max-Pool	2	2	32	(64, 32, 1008)	(64, 32, 504)
	Conv	3	1	64	(64, 32, 504)	(64, 64, 502)
	BN	/	/	/	(64, 64, 502)	(64, 64, 502)
	Conv	3	1	128	(64, 64, 502)	(64, 128, 500)
	BN	/	/	/	(64, 128, 500)	(64, 128, 500)
	Ada-Max-Pool	/	/	/	(64, 128, 500)	(64, 128, 4)
	Reshape	/	/	/	(64, 128, 4)	(64, 512)
	FC	/	/	/	(64, 512)	(64, 256)
	Dropout	/	/	/	(64, 256)	(64, 256)
Fault classifier (Output)	FC	/	/	/	(64, 256)	(64, 128)
	Dropout	/	/	/	(64, 128)	(64, 128)
	FC	/	/	/	(64, 128)	(64, 10)
Distribution discrepancy metrics	FC	/	/	/	(64, 256)	(64, 128)
	Dropout	/	/	/	(64, 128)	(64, 128)
	FC	/	/	/	(64, 128)	(64, 10)

**Table 2:** The diagnostic tasks of CWRU.

Task Code	Speed (rpm)	Load (HP)
0	1730	0
1	1750	1
2	1772	2

**Table 3:** Sample division of six transfer diagnosis tasks.

Transfer Diagnosis Task	Training Dataset		Validation Dataset (Target Domain Data: B)	Testing Dataset (Target Domain Data: C)
	Source Domain Data	Target Domain Data: A		
x − y (x, y ∈ [0, 2]; x, y ∈ N; x ≠ y)	Labeled samples: 1000	Unlabeled samples: 1000	Samples: 300	Samples: 300

**Table 4:** Comparison results of signal preprocessing methods under different transfer diagnosis tasks.

	0-1	0-2	1-0	1-2	2-0	2-1	Average
FFT	99.68 ± 0.49	100.00 ± 0.00	98.98 ± 5.45	100.00 ± 0.00	87.74 ± 6.90	96.39 ± 2.07	97.13
EMD	97.88 ± 3.82	98.20 ± 2.18	94.86 ± 6.66	100.00 ± 0.00	95.17 ± 6.72	98.84 ± 0.73	97.49
VMD	99.71 ± 0.88	99.28 ± 0.51	98.89 ± 0.70	100.00 ± 0.00	96.60 ± 1.68	99.71 ± 0.25	99.03
FFT(4 dB)	97.08 ± 1.95	97.62 ± 1.29	94.38 ± 2.44	99.46 ± 0.37	71.90 ± 5.25	98.05 ± 0.36	93.08
EMD(4 dB)	96.46 ± 0.75	97.11 ± 2.25	94.90 ± 4.04	99.28 ± 0.42	95.32 ± 1.91	97.40 ± 0.36	96.75
VMD(4 dB)	97.32 ± 0.88	97.22 ± 0.90	95.77 ± 1.45	99.28 ± 0.39	96.39 ± 2.16	98.41 ± 0.28	97.40
FFT(0 dB)	92.42 ± 1.79	83.98 ± 2.39	89.14 ± 1.31	96.00 ± 1.59	75.10 ± 5.25	95.56 ± 2.46	88.70
EMD(0 dB)	93.07 ± 1.98	92.35 ± 1.33	94.47 ± 0.32	96.17 ± 1.28	91.92 ± 2.06	94.23 ± 1.36	93.70
VMD(0 dB)	94.30 ± 1.24	97.04 ± 1.48	90.73 ± 3.77	97.91 ± 0.25	94.81 ± 2.67	96.61 ± 1.03	95.24

**Table 5:** Comparison results of different feature fusion methods.

	0-1	0-2	1-0	1-2	2-0	2-1	Average
Concatenate	99.85 ± 0.18	98.84 ± 0.85	97.96 ± 4.98	99.35 ± 0.10	96.94 ± 0.89	99.57 ± 0.50	98.75
Add	99.93 ± 0.28	98.98 ± 5.28	89.20 ± 4.89	99.93 ± 0.19	94.47 ± 7.60	98.92 ± 1.46	96.90
SEA	99.64 ± 3.86	98.41 ± 0.64	97.96 ± 7.42	99.28 ± 0.47	95.58 ± 4.85	99.64 ± 0.59	98.42
ECA	99.71 ± 0.88	99.28 ± 0.51	98.89 ± 0.70	100.00 ± 0.00	96.60 ± 1.68	99.71 ± 0.25	99.03

**Table 6:** Comparison results of the proposed method with other state-of-the-art methods.

	0-1	0-2	1-0	1-2	2-0	2-1	Average
WDCNN	96.62 ± 2.34	94.61 ± 3.35	98.36 ± 0.81	99.98 ± 0.06	97.50 ± 2.25	96.36 ± 1.59	97.24
DDC	98.15 ± 2.06	98.77 ± 1.93	97.79 ± 1.50	100.00 ± 0.00	98.89 ± 3.74	98.48 ± 0.57	98.68
DANN	99.42 ± 1.03	99.20 ± 1.44	99.23 ± 0.18	99.71 ± 0.77	97.79 ± 3.12	95.24 ± 1.64	98.43
DTN	97.45 ± 0.80	95.24 ± 2.36	97.70 ± 1.38	100.00 ± 0.00	98.72 ± 0.56	99.49 ± 0.68	98.10
Proposed	99.71 ± 0.88	99.28 ± 0.51	98.89 ± 0.70	100.00 ± 0.00	96.60 ± 1.68	99.71 ± 0.25	99.03
WDCNN(4 dB)	92.33 ± 3.67	95.41 ± 2.53	96.25 ± 1.43	97.16 ± 0.97	95.02 ± 2.39	95.41 ± 0.96	95.26
DDC(4 dB)	95.17 ± 2.10	98.13 ± 3.75	96.43 ± 0.71	98.56 ± 0.74	95.66 ± 1.59	96.68 ± 2.43	96.77
DANN(4 dB)	93.72 ± 4.11	97.47 ± 4.42	96.60 ± 3.77	99.28 ± 0.59	95.92 ± 1.23	96.68 ± 1.21	96.61
DTN(4 dB)	97.50 ± 0.32	97.46 ± 1.02	97.11 ± 0.93	98.63 ± 2.07	95.49 ± 2.35	96.83 ± 1.16	97.17
Proposed(4 dB)	97.32 ± 0.88	97.22 ± 0.90	95.77 ± 1.45	99.28 ± 0.39	96.39 ± 2.16	98.41 ± 0.28	97.40
WDCNN(0 dB)	93.33 ± 1.64	93.09 ± 2.03	91.90 ± 2.91	95.28 ± 0.65	91.08 ± 3.93	95.19 ± 1.24	93.31
DDC(0 dB)	93.65 ± 2.67	94.08 ± 1.54	94.05 ± 2.52	96.32 ± 1.33	93.03 ± 1.47	93.80 ± 2.70	94.15
DANN(0 dB)	94.44 ± 1.78	95.17 ± 1.80	92.93 ± 2.58	97.55 ± 1.13	92.35 ± 2.22	95.45 ± 1.34	94.65
DTN(0 dB)	92.71 ± 0.85	93.80 ± 2.13	93.11 ± 1.38	96.32 ± 1.01	92.94 ± 1.85	95.02 ± 1.67	93.98
Proposed(0 dB)	94.30 ± 1.24	97.04 ± 1.48	90.73 ± 3.77	97.91 ± 0.25	94.81 ± 2.67	96.61 ± 1.03	95.24

## Data Availability

All data used in the experiments can be downloaded from the following links: http://csegroups.case.edu/bearingdatacenter/pages/download-data-file (accessed on 26 July 2021) (CWRU dataset).
